# Two Cases of Successful Removal of the Upper Jaw, on Account of Malignant Disease of the Antrum

**Published:** 1851-04

**Authors:** Robert L. MacDonnell

**Affiliations:** Montreal.


### ARTICLE XIX.

*Two Cases of Successful Removal of the Upper Jaw, on account of Malignant Disease of the Antrum.* By Robert L. MacDonnell, M. D., &c., of Montreal.

Case I.—Mr. ———, aged 55, a farmer, residing in the Eastern Townships, consulted me in June, 1849, on account of a tumor which occupied the right cheek, and extended into the nostril of the same side. He stated that about a year previous, he had received a blow on the cheek, which at the time caused but little annoyance, but that three months afterwards he began to suffer some pain in the seat of the injury, and he then noticed a small hard tumor, at first scarcely perceptible, but which soon increased in size, and gradually acquired its present dimensions. Within the last few nonths, the tumor became larger and more painful, and extended into the right nostril, and frequent and profuse attacks of epistaxis quickly ensued. His general health began to fail, and he lost all hope of discussing the tumor by poultices and fomentations—the only treatment he had latterly employed. The surgeon to whom he applied in the country, extracted a molar tooth, and explored the cavity of the antrum, but nothing except a few drops of blood escaped. When I saw him, the tumor was about the size of a hen egg, and projected from the canine fossa of the superior maxilla, to which it was firmly adherent.

The skin covering it was tense and shining. The right nostril was blocked up by a fungous growth, which was hard and resisting, of a dark reddish color, and exhibited a great tendency to bleed on being touched. A ridge-like projection was likewise observed above the alveolar process of the same side, but no protrusion through the hard palate was noticed. The eye-ball was not displaced, and no impairment of vision had taken place. His general health appeared good, with the exception of the debility induced by the losses of blood from the nasal portion of the tumor. In consultation with Drs. Campbell, Howard and Crawford, it was resolved to excise the superior maxilla, and accordingly the operation was performed, on the 18th of June, in the following manner:

A short horizontal incision was made along the zygoma to the body of the malar bone, from which point the incision was now carried in a curvilinear manner to the angle of the mouth. The convexity of this incision was directed towards the ear. The flap was then dissected from off the tumor as far as the nose, and its external extent, and that of the maxilla, were thus fully exposed—a natural separation between the incisors rendered the extraction of one of them unnecessary. The alveolar process, hard palate and malar bone were divided by means of a small sharp forceps, and the nasal process of the maxilla was divided with equal ease. An incision was made through the mucous membrane along the median line of the palate, and transversely corresponding to the junction of the palate and maxillary bones, and with a slight wrenching motion the bone and the diseased mass were removed; a few touches of the knife liberated them from any soft adhesions that remained undivided.

The orbital portion of the maxilla was not removed, as it appeared quite healthy. The facial artery was the only vessel that required a ligature, and the amount of blood lost was very trifling.

The flap was laid down, and the edges of the wound brought together by twisted and interrupted sutures; the cavity in the mouth was filled with fine lint, and water dressing was applied externally.

July 21st.—Some of the sutures and needles were removed, and the external wound was found to have united nearly throughout its whole extent, a small portion in its center being still open. The patient was able to sit up and walk about, no collapse having followed the operation. For the next fortnight nothing worthy of notice occurred; and as he was very anxious to return home, he was allowed to do so, a small portion of the external wound remaining still open.

I heard from him several times after his departure from Montreal; in some of his letters he complained of pain in the former situation of the tumor, and of a discharge of a sanious fluid from the nostril. Whilst preparing this for the press, I have applied to his former medical attendant for more accurate information, but have not, as yet, received an answer to my inquiries. He was alive, however, twelve months after the operation, a fact clearly in favor of its performance, for there can be but little doubt that the frequent attacks of hemorrhage would have carried him off in a few months, even if the disease had not produced death by pressure on important parts.

Case II.—J. Wallace, aged 30, but looking much older, of a sallow complexion, tall, and of thin spare habit, was admitted into the Montreal General Hospital, under my care, May 2, 1850. He stated that two years before he noticed a small tumor, of a wart-like nature, growing from the hard palate, which, as soon as it had attained the size of a marble, remained stationary until five months before admission, when it began to spread over the palate, so as to occupy nearly three-fourths of its extent. He also remarked, that a tumor had commenced to form on the left superior maxillary bone, and now, for the first time, he suffered from deep-seated pain in the cheek. He applied to Dr. Lang, of the medical staff at Bytown, who made an exploratory puncture with a small trocar, through the portion of the tumor presenting itself in the palate, but nothing, he says, escaped from the opening. Lunar caustic, in substance and solution, was frequently introduced through the opening, but produced no effect on the size or consistence of the tumor.

On admission, three-fourths of the palate were occupied by a hard tumor, covered by red mucous membrane; on the left side of the face, a large prominence was observed, which, when looked at sideways, nearly obscured the nose; it displaced the nasal bone which lay upon its surface, and extended to the edge of the orbit. A portion of the growth could be seen in the upper part of the left nostril, but no hemorrhage had as yet proceeded from it, nor did he complain of pain when it was touched. The eyesight was not impaired. The articulation was very indistinct, and he had latterly experienced much difficulty in masticating and swallowing his food.

The removal of the bone was performed on the 6th of May, in the following manner, in the presence of the staff of the hospital and several visitors. As the tumor encroached so much upon the nose, and had extended to the palate-plate of the *opposite* maxilla, I considered it more prudent to make two incisions through the integuments, as recommended by Liston; one externally, (as in the first case,) in a curved direction from the malar portion of the zygoma, to the angle of the mouth; the other in a perpendicular direction from the inner angle of the eye, along the side of the nose, as far as its ala; then transversely along the upper portion of the lip, as far as the median fissure, whence it was carried perpendicularly down through the whole thickness of the lip. The flap thus made was dissected upwards, and the tumor and bone fully exposed. The infra-orbital nerve was divided, and the globe of the eye and the inferior oblique muscle carefully dissected from the orbital plate. The mucous membrane was next divided, between the tumor and the alveolar process of the *right* maxilla, and a transverse incision was carried along the palato-maxillary articulation. The incisors having been removed before the operation was commenced, one blade of Liston's forceps was passed into the *right* nostril close to the septum, and the alveolar process and palate plate of the *right* maxillary bone were readily divided. The left malar bone and the nasal process of the left maxillary were next cut through, and the tumor was thus detached, with the exception of a few adhesions of the soft parts, which required the use of the knife.

In this case, chloroform was administered during the first stage of the operation, but the patient was allowed to recover from its effects before the division of the bones was commenced. The facial artery was the only vessel that required tying, and very little blood was lost. The collapse which followed the operation was very great, and required for its treatment a liberal administration of hot wine and diffusible stimulants. The patient became, moreover, extremely desponding, and from both these circumstances, the prognosis was very doubtful for the first forty-eight hours. On the third day, however, he rallied, and was able to permit the wounds to be dressed, which were found to have united by the first intention throughout their whole extent.

From this period nothing important occurred. The cavity filled up rapidly with healthy granulations, and exhibited a marked tendency to contract. His articulation and power of deglutition were greatly improved, and his general health soon became re-established; and on June 5th he left hospital to return to his occupation, which was that of a farmer.

A short time ago I addressed a note to Dr. Lang, to inquire about this patient, to which he politely sent me the following answer:

Bytown, *Dec.* 16, 1850.

Dear Sir:—I must apologize for delaying so long to answer your note; but I have been unable, until this morning, to obtain the intelligence you wished. The individual whose maxilla you removed, is now doing well, and at his daily work. The report I have had is, that *the bone is meeting*. Of course you know what they mean by this. The operation has been perfectly successful.

On examination, both these tumors proved to be malignant; they both presented well-marked examples of encephaloid cancer; and it is not a little curious, that seeing they resembled one another in their minute pathological characters, they should have differed so widely in the symptoms accompanying each.

In the first case the growth was rapid, and the nasal portion of the disease bled freely when touched, and from it profuse hemorrhages had spontaneously proceeded; whereas, in the second case, the tumor was slow in its growth, and, even when punctured with a trocar, exhibited no tendency to hemorrhage, nor did any fungus sprout from the opening thus made—consequences so frequent, that few surgeons like opening into, or otherwise meddling with, such growths. In fact, though the patient's countenance indicated malignant disease, I was in hopes, from the hardness of the tumor, its slow growth, its arising in the *palate-plate first*, its indisposition to bleed, and its not having taken on excessive action, nor thrown out fungous growths, when punctured and injected with caustic, that it would prove to be non-malignant.

In the first case, it was my intention to have made two incisions of the cheek, as in the second instance; but, at the suggestion of Dr. Campbell, I was induced to adopt the plan of the single curved incision, and found no difficulty in exposing the tumor and maxillary bone as far as its junction with the nasal bone.

Mr. Syme has claimed this curvilinear incision as an improvement of his own, in contradistinction to the double incision recommended first by Mr. Liston, and subsequently by Fergusson and Miller.

In Mott's edition of Velpeau's Operative Surgery, it is stated that "Dr. Mott had, many years before that date, (the publication of Mr. Syme's paper in 1829,) adopted the curved incision in question, in his exsections of both the upper and lower jawbones; also, Professor Velpeau was, we believe, anterior to Mr. Syme in this matter. It is inexact, therefore, for Mr. Syme to assert, that in operations, either on the upper or lower jaw, it had hitherto always seemed necessary to make a double incision, so as to permit the formation of a flap exposing the fore part of the bone."—Vol. ii, p. 733. From the above passage, it would appear that Professor Mott lays claim to the curved single incision recommended by Mr. Syme; yet, in the same volume, at page 728, we find the following sentence, in the comments on Dr. O'Shaughnessy's cases: "In his operations on the upper jaw, we perceive that he disapproves of the extensive incisions of Mr. Liston, but, nevertheless, continues upon the erroneous plan, as Dr. Mott conceives it to be, of making his incision extend from the zygoma into the center of the commissure of the mouth, instead of the straight and single incision of Dr. Mott, from near the inner angle of the eye and along the ala of the nose, into the mouth, near the median line of the upper lip." If this latter be Dr. Mott's method, it is certainly different from Mr. Syme's, and therefore his claim to priority cannot be admitted; nor can the claim set up by M. Velpeau be supported, for the first allusion to it is made in his letter to Dr. Mott, dated Paris, August 16, 1843, and published in Mott's edition of his work, in 1847; whereas Professor Syme's paper appeared in *Cormack's Monthly Journal*, in February, 1843.

The practical point deducible from the observations of these eminent surgeons is, that it is by no means necessary to make the double incision in *all* cases; and I have no doubt that the practitioner will meet with some in which he will prefer the curvilinear incision; in others, he will deem Mott's straight incision along the side of the nose most advisable; whilst in a third class, he will find it most convenient to adopt Liston's method.*

* In claiming the discovery of the above method, and dating its origin so far back as 1829, Mr. Syme seems to forget that in the last edition of his System of Surgery, published in February, 1842, exactly one year prior to the appearance of his second paper in *Cormack's Monthly Journal*, he himself recommended *exclusively* the two incisions. These are his directions for the first stage of the operation: "In performing excision of the superior maxillary bone, *two incisions* should be made through the cheek, and extending from the inner angle of the eye directly downwards to the lip, the other beginning over the junction of the maxillary and malar bones, and terminating at the angle of the mouth." (p. 487.) If Mr. Syme really considered his plan so great an improvement as he appears to do in the papers published twelve months after the above sentence was sent before the profession, he must have acquired his experience of its merits in the interval between February, 1842, and February, 1843.

In the foregoing cases, the bones were easily divided by strong cutting pliers; and if the operator possess the requisite strength, he can, in almost every instance, dispense with all other instruments for that purpose; for the section can be performed with comparatively such rapidity, that the sufferings of the patient are thereby much diminished, and the operation completed in much less time than by any other plan. The surgeon should furnish himself with pliers of different sizes; for the malar bone and the nasal process of the superior maxillary can be cut through more conveniently with a small bone-forceps, than with that sold as "Liston's forceps."

P. S. *December* 27, 1850.—Whilst correcting the press, I have received from Dr. S. Macdonald an answer to my letter of inquiry. He states, that the subject of my first case died on the 23d of last October, one year and four months after the operation. A morbid growth commenced to form on the same side of the face, in the month of July, and rapidly increased, and gave rise to profuse discharges of pus, also to slight arterial hemorrhages.—*Brit. Amer. Med. and Phys. Jour.*

